# Molecular characterization of the sequences of the 16S-23S rDNA internal spacer region (ISR) from isolates of *Taylorella asinigenitalis*

**DOI:** 10.1186/1756-0500-2-33

**Published:** 2009-03-03

**Authors:** Akihiro Tazumi, Shinji Ono, Tsuyoshi Sekizuka, John E Moore, B Cherie Millar, Motoo Matsuda

**Affiliations:** 1 Laboratory of Molecular Biology, School of Environmental Health Sciences, Azabu University, Fuchinobe 1-17-71, Sagamihara 229-8501, Japan; 2 School of Biomedical Sciences, University of Ulster, Cromore Road, Coleraine, Co. Londonderry, BT52 1SA, Northern Ireland, UK; 3 Department of Bacteriology, Northern Ireland Public Health Laboratory, Belfast City Hospital, Belfast BT9 7AD, Northern Ireland, UK

## Abstract

**Background:**

Sequence information on the 16S-23S rDNA internal spacer region (ISR) exhibits a large degree of sequence and length variation at both the genus and species levels. A primer pair for the amplification of 16S-23S rDNA ISR generated three amplicons for each of isolates of *Taylorella asinigenitalis *(UCD-1^T^, UK-1 and UK-2).

**Findings:**

Following TA cloning and sequencing, the three isolates of *T. asinigenitalis *were demonstrated to possess three ISR units of different lengths. Although the three corresponding ISRs (A, B and C) were identified to be identical to each other (UK-1 and UK-2 isolates), the ISRs shared approximately 95.3–98.9% nucleotide sequence similarities between the UCD-1^T ^and UK-1/-2 isolates. A typical order of two intercistronic tRNA genes (5'-tRNA^Ile^-tRNA^Ala^-3') with the different nucleotide spacers [44 through 51 base pairs (bp)] in length was identified among the isolates. The consensus sequences of the antiterminators of **boxB **and **boxA **were also identified in all ISRs. Thus, three ISRs were identified for each isolate, and therefore, at least three distinctly different ribosomal RNA operons were suggested to occur in the genome of *T. asinigenitalis*. This was also confirmed by Southern hybridization procedure.

**Conclusion:**

The present study represents a dendrogram constructed based on the nucleotide sequence data of 16S-23S rDNA ISR for *T. asinigenitalis*, which may aid in the phylogenetic positioning of *T. asinigenitalis *within the genus *Taylorella*, and in the molecular discrimination of *T. asinigenitalis*.

## Background

*Taylorella equigenitalis*, a Gram-negative, non-motile coccobacillus β-*Proteobacteria*, is an important pathogen responsible for contagious equine metritis (CEM) [1-4].

In late 1997 and in early 1998, three bacterial isolates were isolated from donkey jacks (*Equus asinus*) in the USA and a new second species of the genus *Taylorella*, *T. asinigenitalis*, was established for these atypical organisms [5,6]. Additional *T. asinigenitalis *isolates (Bd3751/05 and 115/04) were obtained more recently and were identified from the genital tract of stallions in Sweden (GenBank accession No. DQ099547) and in Spain (DQ 393780). Sequences of the nearly full-length 16S rDNA from all these five isolates of *T. asinigenitalis *have already been deposited in DDBJ/EMBL/GenBank (AF297174 for UK-1, AF297175 for UK-2, AF067729 for UCD-1^T^, DQ099547 for Bd3751/05 and DQ393780 for 115/04). It was demonstrated that the sequences were almost identical (> 99.8% similarity) among the three isolates obtained in the USA [6]. However, no sequence information on the 16S-23S rDNA internal spacer region (ISR), which exhibits a large degree of sequence and length variation at both the genus and species levels [7,8], of *T. asinigenitalis *have yet been reported.

Therefore, the aim of the present study is to clone, sequence, and analyze the 16S-23S rDNA ISRs of three isolates of *T. asinigenitalis *(UCD-1^T^, UK-1 and UK-2) and to compare the ISRs sequence data.

## Methods

In the present study, three isolates of *T. asinigenitalis *(UCD-1^T^, UK-1 and UK-2; [5,6]) were analyzed. The conditions for cell culturing have previously been described by Matsuda and colleagues [9,10]. Genomic DNA preparation for the PCR amplification was carried out, as described already [11,12].

The primer pair used for the 16S-23S rDNA ISR amplification of *T. asinigenitalis *isolates in the present study was ISR-Tef (5'-CTGGGGTGAAGTCGTAACAAG-3') for the forward primer and ISR-5r (5'-GCCAAGGCATCCACC-3'; the sequence of region 5 reported by Gurtler and Stanisich (1996) [7,12] for the reverse primer. The ISR-Tef primer was designed *in silico *in the present study for PCR amplification of full-length ISR of the genus *Taylorella*.

PCR mastermix contained 10 mM Tris-HCl (pH 8.3), 50 mM KCl, 1.5 mM MgCl_2_, 40 mM of each dNTP, 1 μM of each primer and 0.5 unit of EX Taq™ DNA polymerase, which possesses 3'-5' exonuclease activity (Takara BioInc. Shiga, Japan). PCR was performed in a 50 μl volume, for 25 cycles at 94°C for 1 min, 65.4°C for 30 sec, 72°C for 30 sec and finally 72°C for 7 min. Purification of the amplified PCR products and TA cloning of the PCR amplicons were carried out, as described previously [12]. Dideoxynucleotide sequencing and sequence analysis were also performed, as described previously [12]. For accurate sequencing, multiple TA-cloned PCR products were sequenced. The sequence data of the 16S-23S rDNA ISR of *T. asinigenitalis *determined in the present study have been deposited in DDBJ/EMBL/GenBank (AB264283–AB264291).

Southern blot hybridization analysis of the ISR was carried out using digoxigenin (DIG) labeled fragment of the ISRA (nucleotide position 452–761:AB264283) prepared from the *T. asinigenitalis *UCD-1^T^, as a probe with *Pst *I digested whole genome DNAs, respectively, according to the procedure described by Sambrook *et al*. (2001) [11]. Random primer extension was performed in order to prepare the fragment using a DIG-High Prime kit (Roche Applied Science, Penzberg, Germany).

Nucleotide sequences of the ISRs from the isolates of the genus *Taylorella *were compared to each other using CLUSTAL W software [13], which was incorporated in the GENETYX version 9 (GENETYX Co., Tokyo, Japan) computer software. Following this, a phylogenetic tree was constructed by an unweighted pair group mean average (UPGMA) method available on the GENETYX (version 9).

## Results and discussion

**Figure 1:**
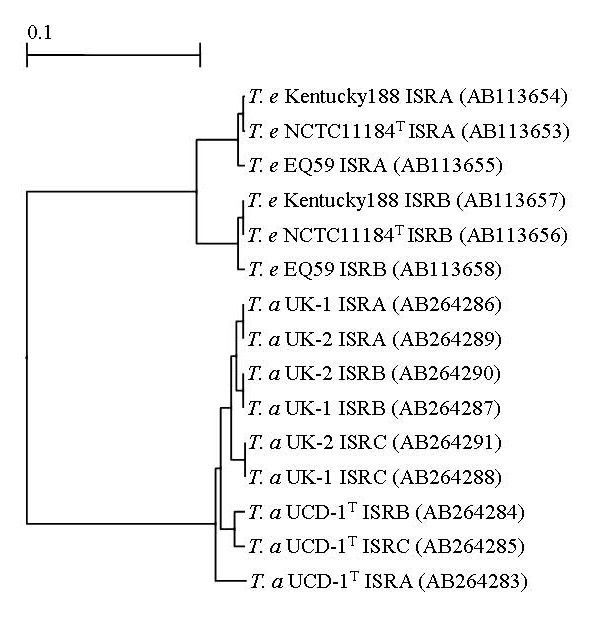
**A phylogenetic tree constructed based on the nucleotide sequence information of the ISRs, by using the UPGMA method**. Values, 0.1, in the figure represent evolutionary distances.

The PCR primer pair (ISR-Tef and ISR-5r) used for the amplification of the segments containing the 16S-23S rDNA ISRs of the three *T. asinigenitalis *isolates produced three amplicons of approximately 930 to 1,050 bp in length for the three isolates, respectively (data not shown). This may imply that *T. asinigenitalis *possesses at least three ISR units of different lengths. Subsequently, nine purified and cloned fragments, containing the ISRs, were subjected to TA cloning and sequencing. The sequences corresponding to the PCR primers (ISR-Tef/-5r) were excluded from the sequence data and further analysis in the present study. The sequences containing the two 16S-23S rDNA ISRs from *T. equigenitalis *NCTC11184^T ^(AB113653 and AB113656) were also compared. The genetic relatedness of the ISRs is shown in Figure 1.

The sequence corresponding to the CCUCCU sequence, a highly conserved sequence close to the 3' end of the mature 16S rRNA that is complementary to the Shine-Dalgarno sequence on mRNA [14,15], was identified in the sequences of the amplicons of *T. asinigenitalis *isolates [nucleotide position 31–36; AB264283–AB264291], as well as *T. equigenitalis *isolates (AF408197, AB066372, AB069660, AB113653–AB113658). Thus, in the present sequencing study of the ISRs of *T. asinigenitalis*, CCTCCT sequences were observed in all isolates examined, where the 3' end of some bases downstream of the CCTCCT sequence may represent the probable 3' end of the 16S rRNA gene.

**Figure 2:**
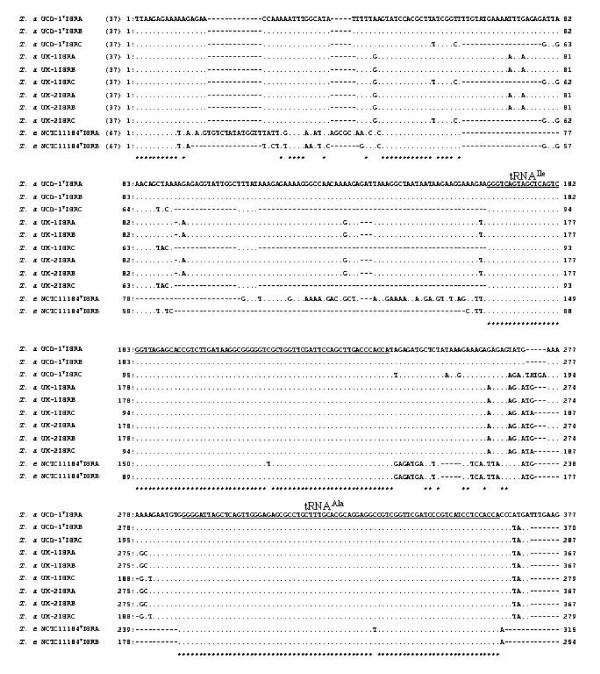
**Nucleotide sequence alignments of the reagion including two intercistronic tRNA genes in the ISRs from the three isolates of T. asinigenitalis**. Reference sequences of the ISR-A and -B from the NCTC11184^T ^of *T. equigenitalis *are also shown for a comparison. Dots indicate identical bases; changes are explicitly indicated; positions identical in all isolates are marked by asterisks; two tRNA genes, for tRNA^Ile ^and tRNA^Ala^, are underlined. Numbers at the left and right refer to nucleotide positions of the ISRs. Numbers in parentheses are the nucleotide positions accessible in the DDBJ/EMBL/GenBank (AB264283 – AB264291).

In relation to the 3' end of the ISR of *T. asinigenitalis *isolates, the probable 3' end of the ISR may represent the nucleotide position 989 of the NISR-A of *T. equigenitalis *NCTC11184^T^, since the nucleotide position 990 of the NISR-A of *T. equigenitalis *NCTC11184^T ^[12] was previously deduced to be the possible 5' end of the 23S rDNA sequence of *T. equigenitalis *by the nucleotide sequence alignment and analysis of the ISR sequences, with the 5' end sequences of the 23S rDNA from several bacterial species (see Figure 2 in the paper described by Kagawa *et al*. (2006) [12].

Consequently, the 16S-23S rDNA ISRs (A, B and C) from the three isolates of *T. asinigenitalis *were estimated to be approximately 837 to 955 bp. The ISR sequence data characteristically indicate that the three corresponding ISRs (A, B, and C) were identified to be identical to each other (UK-1 and UK-2 isolates). In addition, the ISR sequences shared nucleotide sequence similarities of approximately 95.3–98.9% between UCD-1^T ^and UK-1/UK-2 isolates, respectively.

In addition, as shown in Figure 1, a dendrogram constructed, based on the nucleotide sequence information of the ISRs, demonstrated that *T. asinigenitalis *isolates formed a major cluster separating from *T. equigenitalis *isolates (Fig. 1). Thus, nucleotide sequence divergence with 16S-23S rDNA ISR for *T. asinigenitalis *could be useful to discriminate among the isolates and between species within the genus *Taylorella*.

**Figure 3:**
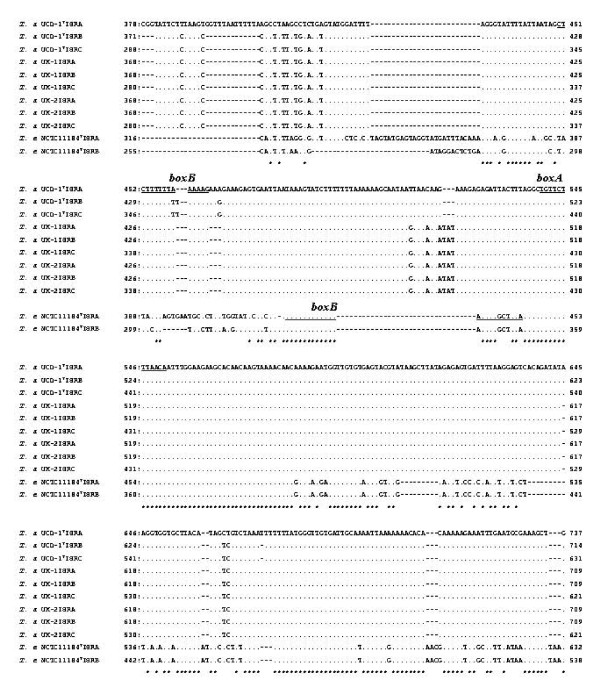
**Nucleotide sequence alignments of the region including the consensus sequence of the antiterminators of boxB and boxA in the ISRs from the three isolates of T. asinigenitalis and T. equigenitalis NCTC11184^T ^isolate**. The regions of **boxB **and **boxA **are underlined. For other details, refer to the Figure 2.

A typical order of intercistronic tRNA genes with 44 through 51 nucleotide spacers of 5'-16S rDNA-tRNA^Ile ^(nucleotide position 202–278 of the ISR-A of UCD-1^T^) -tRNA^Ala ^(nucleotide position 325–399 of the ISR-A of UCD-1^T^) -23S rDNA-3' was identified in all three kinds of ISRs from the three *T. asinigenitalis *isolates. Figure 2 clearly demonstrates, that both genes for tRNA^Ile ^and tRNA^Ala ^were almost identical among the ISRs from *T. asinigenitalis*, as well as *T. equigenitalis *(Fig. 2). The consensus sequences of the antiterminators of **boxB **(a stem-loop structure with no apparent sequence conservation at nucleotide position 486–500) and **boxA **(a conserved sequence, TGTTCTTTAACA, at nucleotide position 576–587 in AB264283 for the ISR-A of UCD-1^T^) were also identified in all three kinds of ISRs (Fig. 3). As illustrated in Figure 3, **boxA **occured with sequences among the 11 ISRs from the genus *Taylorella *examined. Although the nucleotide sequence differences in the **boxB **were demonstrated at two loci between the UCD-1^T ^ISR-A and -B/-C in the 15–16 nucleotide sequence, **boxB **of UCD-1^T ^ISR-A was identical to those of UK-1 and -2. In addition, the two ISR-A and -B from *T. equigenitalis *NCTC11184^T ^were relatively divergent with respect to **boxB **(nucleotide position 487–509 in AB113653 for the ISR-A) from those of *T. asinigenitalis *isolates, respectively, as shown in Figure 3.

**Figure 4:**
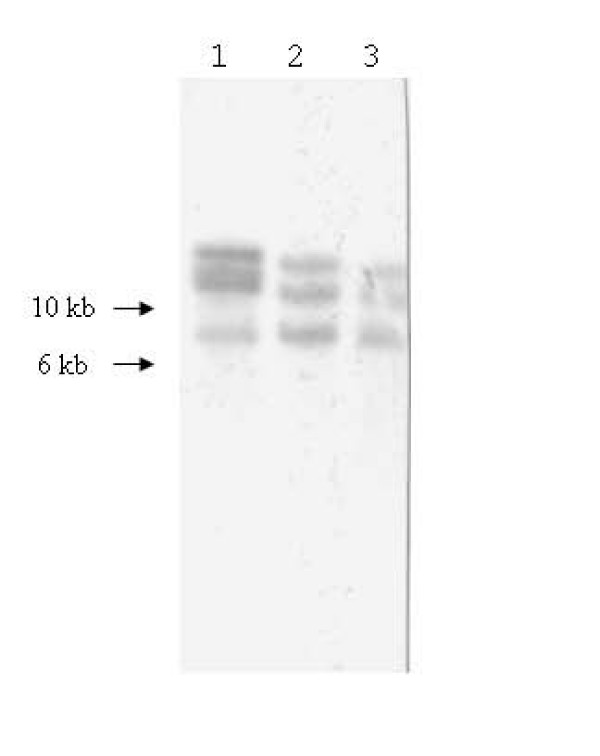
**Southern blot hybridization analysis of genomic DNA digested with Pst I from T. asinigenitalis UCD-1^T ^(lane 1), UK-1 (lane 2) and UK-2 (lane 3) isolates, by using the ISRA from the UCD-1^T ^(UCD-1^T^A) as a probe**.

When Southern blot hybridization analysis was carried out in order to clarify how many ISRs occur in the genome of the three *T. asinigenitalis *isolates, the occurrence of the three was identified in all the three isolates, respectively (Fig. 4).

Consequently, in the present study, it was demonstrated that three isolates of *T. asinigenitalis *possess three kinds of ISRs with different length, respectively. Thus, the present results may possibly indicate that *T. asinigenitalis *isolates carry at least three distinctly different ribosomal RNA operons in the genome.

Jang *et al*. (2001) previously demonstrated that pulsed-field gel electrophoresis (PFGE) profiles after digestion with *Not *I of the genomic DNAs from the two isolates (UK-1 and UK-2) of *T. asinigenitalis *were the same, but they differed from the other isolate (UCD-1^T^) [6]. The present sequencing results of the 16S-23S rDNA ISR (A, B and C) clearly identified that the three ISRs were quite identical to each other [UK-1 and UK-2], but they were different from those of the UCD-1^T^, respectively. Thus, our present sequence informations of the ISRs are consistent with the result of PFGE analysis by Jang *et al*. (2001) [6]. The two isolates (UK-1 and UK-2) of *T. asinigenitalis *obtained from donkey jacks in Kentucky, USA, in 1998 [6] gave the quite identical profiles of the PFGE and the 16S-23S rDNA ISR sequences. Therefore, these two isolates may possibly be identical.

Overall, this study is the first report of 16S-23S rDNA ISR sequences and tRNA genes from *T. asinigenitalis*. This is also first molecular comparison of the ISRs from *T. asinigenitalis *with those from *T. equigenitalis*.

## Abbreviations

*T*: *Taylorella*; ISR: internal spacer region; PCR: polymerase chain reaction; PFGE: pulsed-field gel electrophoresis.

## Competing interests

The authors declare that they have no competing interests.

## Authors' contributions

MM participated in design of the study, collected isolates, drafted the manuscript and review of the manuscript. AT, SO and TS were involved with cloning, sequencing and analysis of the ISRs from *T. asinigenitalis *isolates. JEM and BCM participated in its design and coordination, and review of the manuscript. All authors have read and approved the final manuscript
